# Integrated analysis of tumor mechanical microenvironment-based signature reveals prognostic risk and immune landscape in endometrial carcinoma

**DOI:** 10.1016/j.gendis.2026.102039

**Published:** 2026-01-13

**Authors:** Yue Qi, Yuman Wu, Xinyi Bi, Xiaoyang Wang, Yiqin Wang, Jingyuan Wang, Jianliu Wang, Xingchen Li

**Affiliations:** Department of Obstetrics and Gynecology, Peking University People's Hospital, Beijing 100044, China

Endometrial carcinoma (EC) is the most common gynecologic malignancy, with increasing incidence and poor prognosis in advanced cases.^1^ While the tumor mechanical microenvironment (TMME) plays a pivotal role in cancer progression, its role in EC remains poorly understood. Here, we developed a TMME-based prognostic model and investigated how glucose metabolism disorders affect the tumor microenvironment, providing new insights into EC prognosis and therapy.

The TMME encompasses biomechanical factors such as extracellular matrix (ECM) remodeling, stiffness sensing, and mechanotransduction, all of which influence tumor growth, invasion, and immunity. Consensus clustering of 530 TCGA-EC samples identified two TMME subtypes (Fig. 1A–D). Kaplan–Meier curves showed that Cluster 1 had significantly poorer overall survival (OS) compared to Cluster 2 (Fig. 1E). Immune profiling revealed higher stromal scores and lower immune infiltration in Cluster 1 (Fig. 1F). Weighted gene co-expression network analysis (WGCNA) identified nine modules, with the turquoise module exhibiting the strongest correlation with mechanical features (Fig. S1A and S1B). Furthermore, cluster 1 showed higher PD1/PD-L2 expression, while Cluster 2 displayed increased CTLA4/CD86 levels (Fig. S1C and S1D).

**Figure 1:**
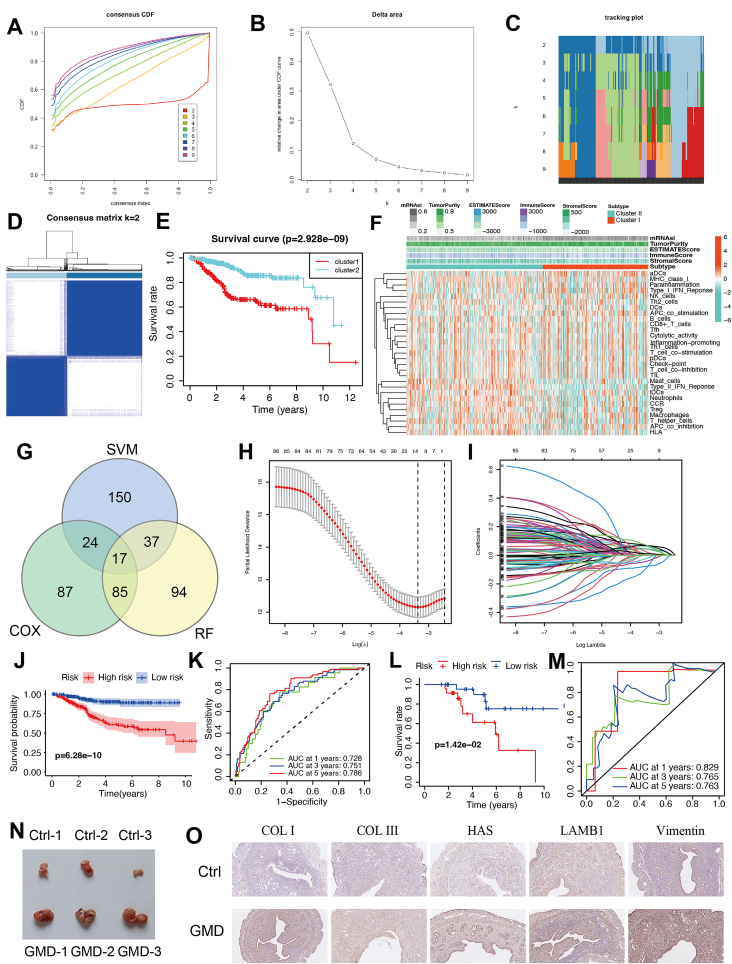
Identification and clinical validation of TMME subtypes and the 12-gene prognostic signature in EC. **(A)** CDF curves for consensus clustering at each K. **(B)** Delta area plot showing relative change in CDF area. **(C)** Sample tracking plot for K = 2–9. **(D)** Consensus heatmap for K = 2. **(E)** Kaplan–Meier curves of two TMME clusters; Cluster 1 (red) shows poorer OS (log-rank *P* < 0.001). **(F)** Immune infiltration and mRNAsi scores of clusters by ssGSEA. **(G)** Venn diagram of overlapping genes identified by SVM, Cox, and random forest. **(H)** LASSO tuning parameter (λ) selection by 10-fold cross-validation. **(I)** LASSO coefficient profiles. **(J)** Kaplan–Meier OS curves for high- and low-risk TCGA groups (95% CI). **(K)** 1-, 3-, and 5-year ROC curves for OS prediction in the TCGA-UCEC cohort. **(L)** Kaplan–Meier OS curves for high- and low-risk PKUPH patients (95% CI). **(M)** 1-, 3-, and 5-year ROC curves for OS prediction in the PKUPH cohort. **(N)** Tumor volumes in mice with glucose metabolic disorders (GMD) *vs* controls (Ctrl). **(O)** IHC comparison of stromal components (COL I, COL III, HAS, LAMB1, Vimentin) between the two mouse groups.

By integrating SVM, Cox, and Random Forest algorithms, 17 key TMME-related genes were initiallyidentified. Subsequently, 12 core genes (*P2RX4*, *SMARCA4*, *C11orf52*, *MMP9*, *KCNJ12*, *LRRC8D*, *NR6A1*, *DIRC3*, *DRD2*, *DHCR24*, *CDKN2A*, and *SIX1*) were selected via LASSO regression to construct the prognostic model (Fig. 1G–I). Kaplan–Meier analysis confirmed shorter OS in the high-risk groups (Fig. 1J), with strong predictive performance for 1-, 3-, and 5-year OS (Fig. 1K). Further validation in the PKUPH cohort (Table S1) showed AUCs of 0.829, 0.765, and 0.763 (Fig. 1L and M). Clinical correlation analysis indicated significant disparities in the distribution of clinicopathologic factors and TMME gene expression across diferent risk strata (Fig. S2).

Univariate and multivariate Cox analyses confirmed the TMME-based signature as an independent prognostic factor (Fig. S3A and S3B). A nomogram integrating independent predictors effectively estimated OS (Fig. S3C). ROC and Kaplan–Meier analyses in the TCGA and PKUPH cohorts demonstrated high predictive accuracy (Fig. S3D–S3H).

Given evidence linking glucose metabolism to ECM remodeling, we established a glucose metabolic disorder (GMD) mouse model (Fig. S4A–S4C). Mice with GMD developed larger tumors (Fig. 1N) and showed ECM stiffening with elevated COL I, COL III, HAS, LAMB1, and Vimentin (Fig. 1O; Fig. S4D). Transcriptomic analysis indicated enrichment of the ECM and PI3K-Akt signaling pathways (Fig. S4E–S4G), suggesting that metabolic dysfunction drives tumor progression through mechanical remodeling. The GMD group exhibited persistent hyperglycemia and reduced weight gain, consistent with insulin resistance models.

Mechanistically, the 12 model genes represent complementary functions within the TMME. MMP9 promotes ECM degradation; P2RX4 and KCNJ12 regulate mechanosensitive ion flux; SMARCA4 and LRRC8D mediate biomechanical and osmotic responses; DIRC3 and DRD2 modulate cytoskeletal dynamics via RhoA/ROCK; DHCR24 affects membrane stiffness and redox balance; CDKN2A and NR6A1 regulate the cell cycle under stress; and SIX1 enhances EMT and ECM synthesis. All genes originated from the turquoise WGCNA module, which were functionally enriched in ECM organization, focal adhesion, cytoskeletal regulation, PI3K-Akt, and Hippo-YAP/TAZ pathways.

Studies have demonstrated that diabetic patients have a higher risk of developing EC.^2^ Our data further indicate that hyperglycemia-induced ECM remodeling strengthens mechanical stiffness, promoting tumor motility. Firstly, *in vitro* experiments have demonstrated that disorders in glucose metabolism could result in alterations of extracellular matrix components such as COL I and COL III. They represent the crucial components of the extracellular matrix.^3^ Immunohistochemistry (IHC) results confirmed the up-regulation of α-SMA and LAMB1, a laminin subunit essential for nuclear integrity, proliferation, and DNA repair^4^^,^^5^. The overexpression of α-SMA was also a biomarker of cancer metastasis. The alterations of the extracellular matrix illustrated that the stiffness of the matrix may promote the motility of EC cells. Besides, Kyoto Encyclopedia of Genes and Genomes (KEGG) enrichment analysis revealed that GMD may contribute to the mechanical stimulus alternation via the PI3K-Akt signaling pathway. The GMD group showed persistent hyperglycemia and reduced weight gain (Fig. S4A and S4B). Although insulin and lactate were not measured, STZ and high-fat feeding are well documented to induce insulin resistance and lactate accumulation in previous studies.

Compelling evidence demonstrates that the TMME scoring system has significant predictive value for survival, offers comprehensive oncogenic characterization, and effectively stratifies patients for immunotherapy. Notably, our study differs from prior TMME research in other cancers (*e.g.*, breast and colorectal cancers) in two key aspects: (1) While mechanical stiffness in breast cancer primarily drives metastasis via EMT, EC progression in our model is more tightly linked to glucose metabolism-induced ECM remodeling and PI3K-Akt axis; (2) Unlike colorectal cancer where TMME genes regulate angiogenesis, EC-specific genes such as *P2RX4* and *SMARCA4* identified here are involved in calcium signaling and chromatin remodeling, respectively.

Immune profiling revealed significant heterogeneity between TMME subtypes, with Cluster 1 displaying elevated immune checkpoints but reduced cytotoxic infiltration. ECM stiffening and fibrosis may impede immune cell trafficking and foster immunosuppressive cytokine secretion (TGF-β, IL-10, CCL2), promoting regulatory T cells (Tregs) and M2-polarized macrophages while suppressing cytotoxic T cells. Furthermore, hyperglycemia and lactate accumulation may further enhance immunosuppression, linking metabolic stress and immune evasion.

In conclusion, the study demonstrates the predictive value of a TMME-based risk score model for EC prognosis and underscores the importance of glucose metabolism disorders in the modulation of the tumor mechanical microenvironment. The findings highlight potential therapeutic targets, including ECM-related molecules, and pave the way for future research focused on the interplay between metabolism and mechanical forces in EC.

## CRediT authorship contribution statement

**Yue Qi:** Writing – original draft. **Yuman Wu:** Data curation. **Xinyi Bi:** Validation. **Xiaoyang Wang:** Validation. **Yiqin Wang:** Funding acquisition. **Jingyuan Wang:** Methodology. **Jianliu Wang:** Writing – review & editing. **Xingchen Li:** Writing – review & editing.

## Ethics declaration

This research has obtained ethical approval by the Ethics Committee of Peking University People's Hospital (No. 2022PHB397-001).

## Funding

This study is supported by the Major Projects of National Science and Technology (No. 2025ZD0545900, 2025ZD0545901), Research and Development Fund of Peking University People’s Hospital (No. RDJP2025-07), the Peking University Clinical Scientist Training Program (China) (No. BMU2025PYJH007) and the Fundamental Research Funds for the Central Universities, and the Peking University Medicine Fund of Fostering Young Scholars’ Scientific & Technological Innovation (China) (No. BMU2025YFJHPY024).

## Conflict of interests

There is not conflict of interests.

## References

[1] Bo L., Wang Y., Feng Y. (2026). Fertility-preserving treatment of endometrial cancer and endometrial atypical hyperplasia for patients with metabolic abnormalities: challenge or opportunity?. Chin Med J (Engl).

[2] Peeri N.C., Bertrand K.A., Na R. (2025). Understanding risk factors for endometrial cancer in young women. J Natl Cancer Inst.

[3] Na J., Tai C., Wang Z. (2025). Stiff extracellular matrix drives the differentiation of mesenchymal stem cells toward osteogenesis by the multiscale 3D genome reorganization. Biomaterials.

[4] de Leeuw R., Gruenbaum Y., Medalia O. (2018). Nuclear lamins: thin filaments with major functions. Trends Cell Biol.

[5] Rzepecki R., Gruenbaum Y. (2018). Invertebrate models of lamin diseases. Nucleus.

