# Supplementary material for: Integrated analysis of tumor mechanical microenvironment-based signature reveals prognostic risk and immune landscape in endometrial carcinoma
Source: Genes Dis. 2026 Jan 13;13(6):102039. doi: 10.1016/j.gendis.2026.102039 (PMC13380096; doi:10.1016/j.gendis.2026.102039)
Supplement: Multimedia component 1 [file mmc1.docx]

**Supplement data**


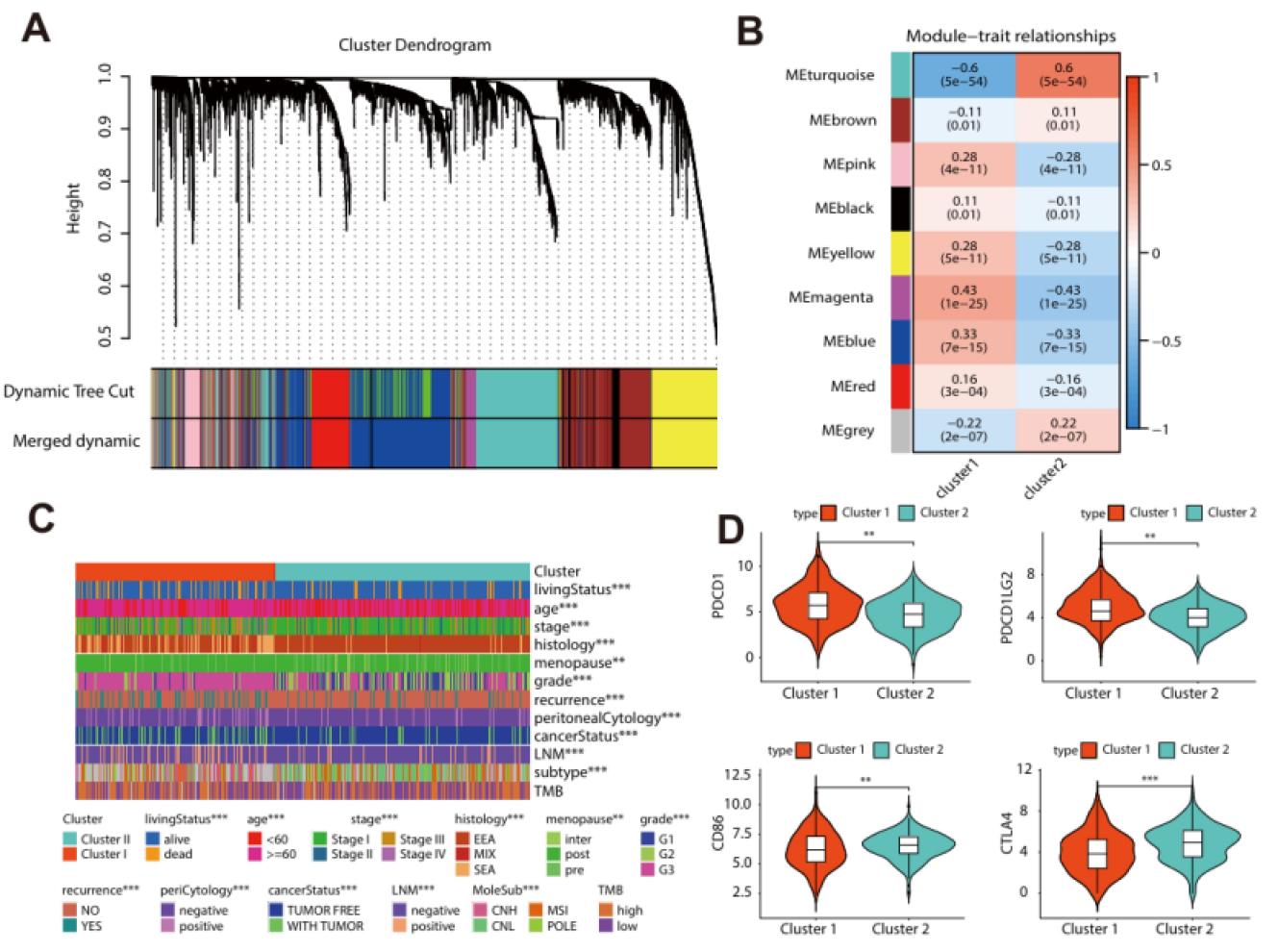


**Figure S1: TMME-associated gene clusters and gene module in EC.** (A) Cluster tree showing that after hierarchical clustering and module merging of the genes, 9 co-expression modules with different colors were obtained;(B) Heatmap of the correlation between module eigengenes and molecular subtypes of EC; (C) Heatmap showing the association between TMME clusters and various clinicopathological features;(D) Violin plots showing the expression levels of immune checkpoints (PDCD1, PDCD1LG2, CD86, and CTLA4) between the two TMME clusters.


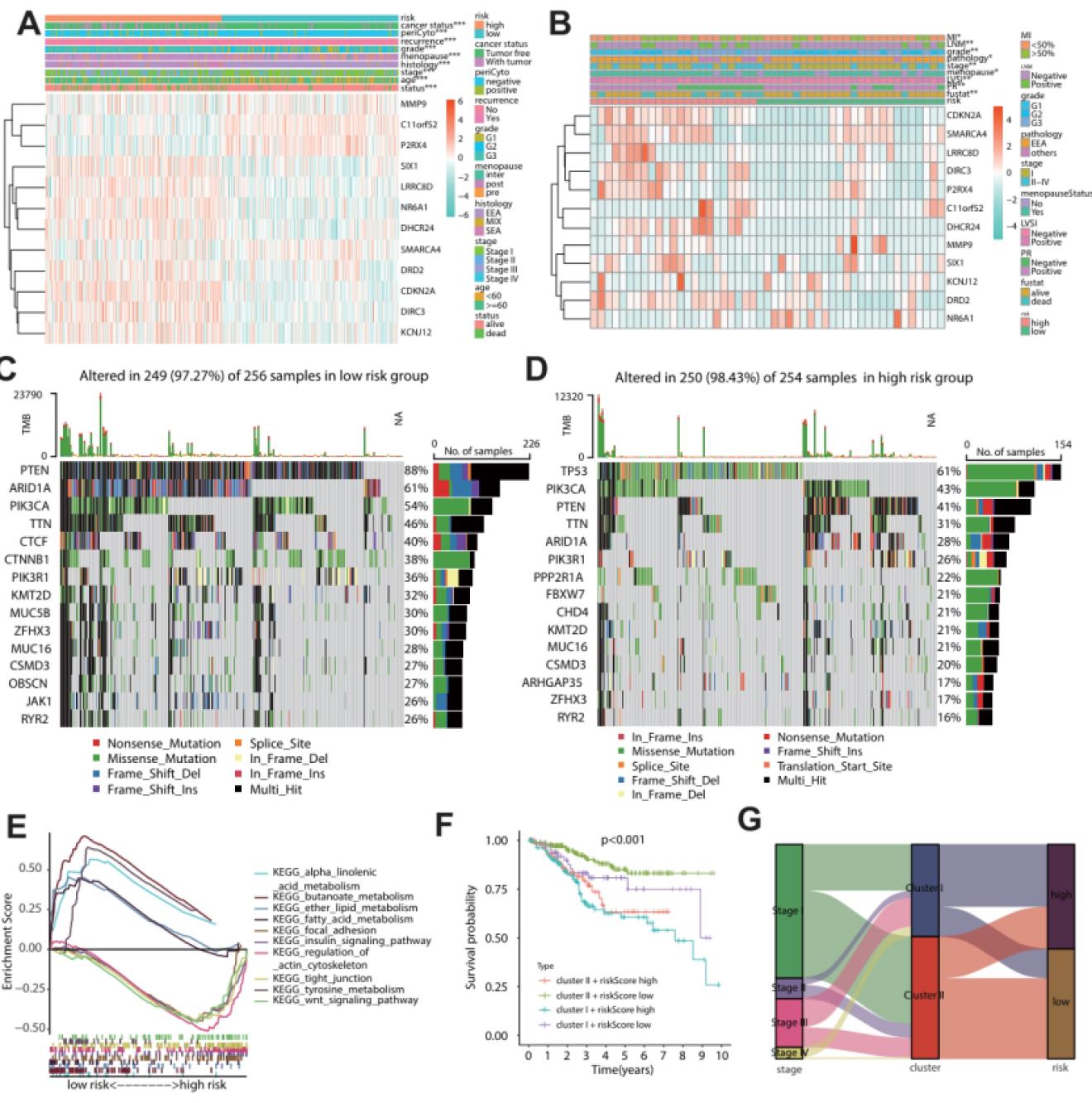


**Figure S2: Correlation of risk models with clinical characteristics based on TCGA and PKUPH cohort.** (A) Heatmap of 12-gene expression in TCGA and PKUPH (B) cohorts. Red indicates high expression, blue indicates low expression (color key scale: -6 to 6); (C) The waterfall plot of tumor somatic mutation established by those with patients in low risk group and (D) high-risk group; (E) GSEA analyses for TMME of two risk signatures; (F) Kaplan‐Meier curves of OS for patients with EC in different clusters and risk signature subgroups; (G) Sankey diagram depicting correlations among FIGO stage, molecular subtypes, and TMME risk signature.


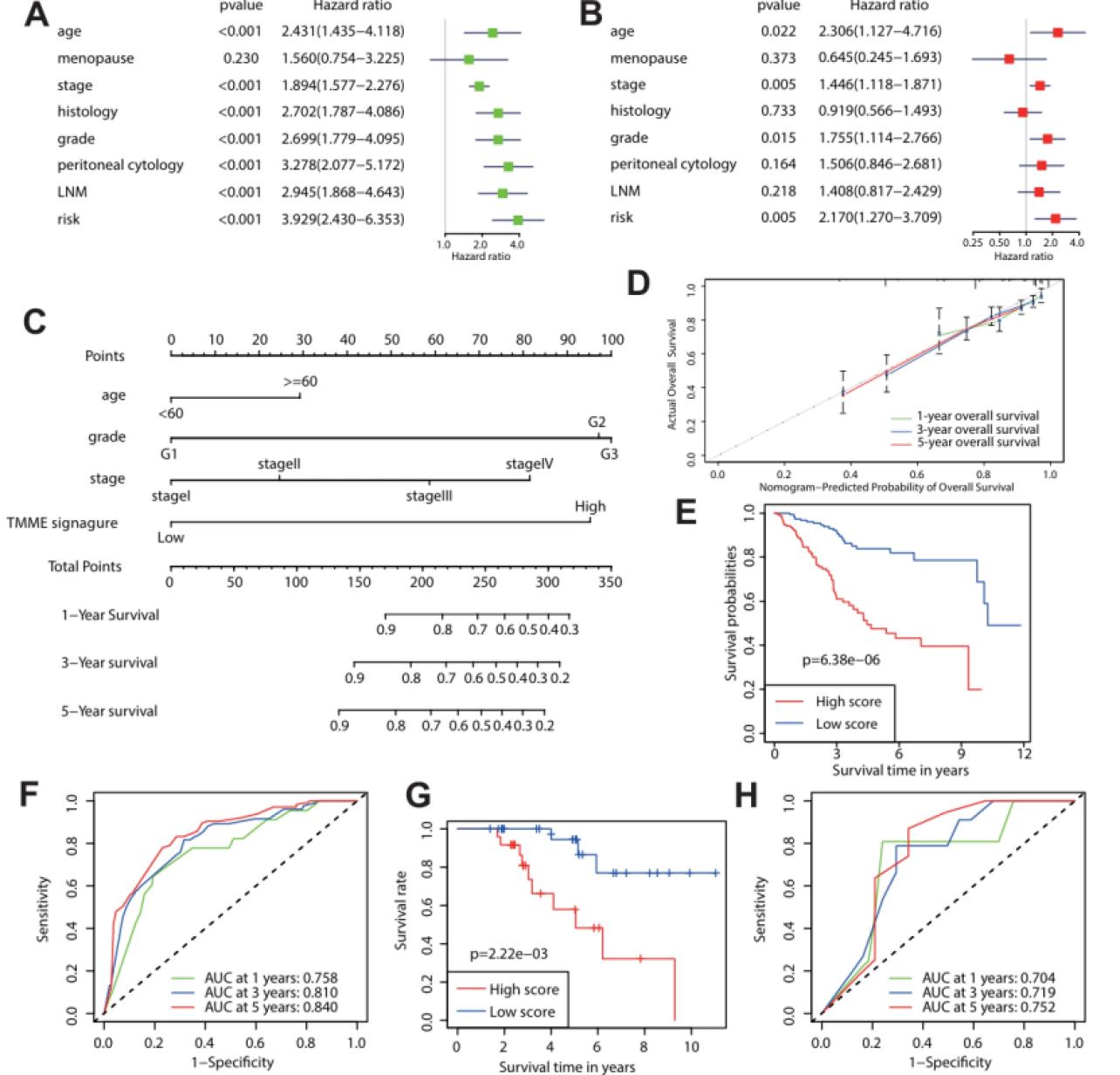


**Figure S3: Development and validation of nomogram model combined with TMME-related risk score and clinical parameters.** (A-B) Univariate and multivariate Cox regression analysis in TCGA cohort; (C) Nomogram integrated the age, grade, stage, and TMME signature; (D) Calibration curves of nomogram; (E) Survival curve with low- and high-score subgroups in TCGA cohort; (F) ROC curve for evaluating 1-, 3-, 5-year survival with nomogram score with TCGA cohort; (G-H) Validation of survival curve and ROC curve in PKUPH cohort.


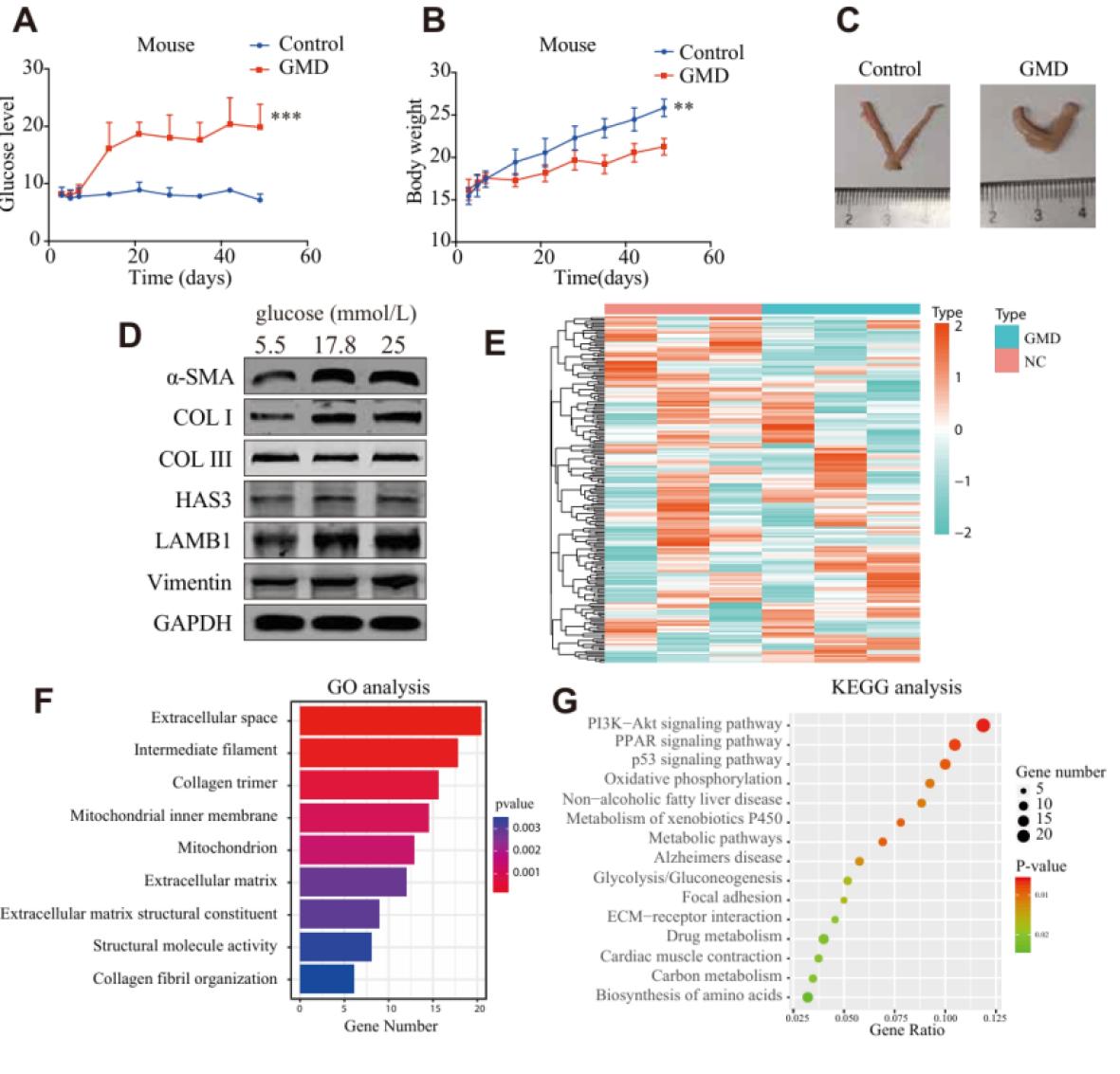


**Figure S4:Experimental validation of mechanism derived from metabolism caused tumor mechanical microenvironment change.** (A) Construction of a metabolic disorder mouse model, with changes in blood glucose levels over time in mice;(B) Changes in mouse weight over time; (C) Changes in uterine morphology in glucose metabolism disorder mouse model and control group mice. (D) Comparison of the expression of matrix main components with different glucose concentrations using Ishikawa cells by Western blot;(E) Heatmap of differentially expressed genes (DEGs) resulting from RNA sequencing in control and metabolism disorder groups;(F) GO and (G) KEGG analysis of DEGs.

Supplementary Table S1:Correlation of risk models with clinical characteristics based on TCGA and PKUPH cohorts

| **GROUP** | **TCGA**  Mean+SD / N(%) | **PKUPH**  Mean+SD / N(%) | **P-value** |
| --- | --- | --- | --- |
| Number | 530 | 49 |  |
| Age (year) | 64.2 ± 11.0 | 61.3 ± 8.5 | 0.182 |
| Survival time | 1172.9 ± 904.7 | 1269.1 ± 890.1 | 0.104 |
| Status |  |  | 0.111 |
| Alive | 438 (82.6%) | 36 (73.5%) |  |
| Dead | 92 (17.4%) | 13 (26.5%) |  |
| MENOPAUSE |  |  | 0.622 |
| Pre-menopausal | 73 (13.8%) | 8 (16.3%) |  |
| Post-menopausal | 457 (86.2%) | 41 (83.7%) |  |
| Stage |  |  | 0.238 |
| I | 332 (62.6%) | 29 (59.2%) |  |
| II | 51 (9.6%) | 9 (18.4%) |  |
| III | 119 (22.5%) | 8 (16.3%) |  |
| IV | 28 (5.3%) | 3 (6.1%) |  |
| Histological type |  |  | 0.091 |
| EEA | 397 (74.9%) | 42 (85.7%) |  |
| Others | 133 (25.1%) | 7 (14.3%) |  |
| Grade |  |  | 0.263 |
| G1 | 97 (18.3%) | 12 (24.5%) |  |
| G2 | 118 (22.3%) | 14 (28.6%) |  |
| G3 | 315 (59.4%) | 23 (46.9%) |  |
| Peritoneal cytology |  |  | 0.375 |
| Negative | 456 (86.0%) | 40 (81.7%) |  |
| Positive | 74 (14.0%) | 9 (18.3%) |  |
| LNM |  |  | 0.093 |
| Negative | 449 (84.7%) | 37 (75.5%) |  |
| Positive | 81 (15.3%) | 12 (24.5%) |  |
