# Supplementary material for: Integrated analysis of tumor mechanical microenvironment-based signature reveals prognostic risk and immune landscape in endometrial carcinoma
Source: Genes Dis. 2026 Jan 13;13(6):102039. doi: 10.1016/j.gendis.2026.102039 (PMC13380096; doi:10.1016/j.gendis.2026.102039)
Supplement: Multimedia component 2 [file mmc2.docx]

**Materials and methods**

**Data Acquisition and Consensus Clustering Using Mechanical Force-Related Differentially Expressed Genes**

To characterize mechanical properties of tumors, we first curated a gene set from the Gene Ontology term “GO_RESPONSE_TO_MECHANICAL_STIMULUS” available in the Molecular Signatures Database (MSigDB, https://www.gsea-msigdb.org/gsea/msigdb/

). This set includes 209 genes involved in ECM organization (e.g., COL1A1, MMP9), cytoskeletal remodeling (e.g., ACTA2, VIM), and mechanotransduction signaling (e.g., YAP1, ITGB1), which have been experimentally validated in prior studies to mediate responses to mechanical stress in cancer and stromal cells.We obtained patient clinical and gene expression data from the TCGA database, from which we extracted 209 genes associated with mechanical stimulation (GO_RESPONSE_TO_MECHANICAL_STIMULUS file, derived from the the gene set enrichment analysis (GSEA) database https://www.gsea-msigdb.org/gsea/msigdb/).Subsequently, cases were divided or condensed into several different clusters based on the provided signatures or features using the consensus clustering analysis method. Additionally, signature gene sets generalize and represent specific, well-defined biological states or processes and show coherent expression. Specifically, we used the consensus clustering algorithm with 1,000 iterations by sampling 80% of the data in each iteration. The optimal number of clusters was confirmed by the Item-Consensus plot, the proportion of ambiguous clustering (PAC) algorithm, and the relative change in the area under the cumulative distribution function (CDF) curve. Two clusters were selected to assess mechanical force differences, and Kaplan-Meier plots were performed for both clusters to compare their overall survival (OS).

**Analysis of immune differences between the two clusters and the Weighted Gene Co-expression Network Analysis (WGCNA) method was employed to identify key gene sets.**

By estimating the differences in stromal and immune cell infiltration in malignant tissues using the ESTIMATE algorithm in the R package IOBR, we obtained immune cell, stromal cell, and ESTIMATE scores for each TCGA-COAD sample (including immune immune score, stromal stromal score, stem cell mRNAsi score, cell purity). To further analyze the data, differential heatmaps of immune cell distribution were generated using the R package "pheatmap." To assess immune cell infiltration and calculate the abundance of each sample's 22 immune cell types, the R package IOBR (v 0.99.9) was utilized, employing the " Cell-type Identification by Estimating Relative Subsets of RNA Transcripts (CIBERSORT)" method (http://cibersortx.stanford.edu/). Furthermore, the expression status of common immune checkpoints between high- and low-risk groups was analyzed by drawing box plots.

The WGCNA method was employed to identify key genes enriched by clusters, and the turquoise set was identified. The selected gene set was then subjected to analysis using the clusterProfiler R software package (v 3.14.3), which provides information on the Gene Ontology (GO) and the Kyoto Encyclopedia of Genes and Genomes (KEGG). False discovery rate (FDR) < 0.05 was considered statistically significant.

**To compare the clinical characteristics as well as the prognosis of the two groups of patients with different TMME subtypes.**

The clinical characteristics of patients in the TCGA database were analyzed to compare survival status, FIGO stage, grading, recurrence, and lymph node metastasis between the two subtypes. A comparative analysis of the expression of immunotherapy-related genes was also conducted.

**Machine Learning to Identify Key Genes Associated with Mechanical Forces and Affecting Prognosis**

To identify key genes associated with mechanical forces and prognosis, we applied an integrative multi-algorithm strategy. Initially, 209 mechanical stimulus–related genes were analyzed using three independent machine learning methods: SVM-RFE, Random Forest, and univariate Cox regression. Genes with a variable importance score > 0.8 in SVM-RFE, a mean decrease in Gini index ≥ 2.0 in the Random Forest model, and a p value < 0.05 in the Cox regression were retained. The intersection of the three algorithms yielded 17 overlapping candidate genes. Subsequently, LASSO Cox regression was performed to minimize overfitting and select the most prognostically relevant variables, resulting in a final set of 12 TMME-related genes for model construction.The optimal tuning parameter (λ) was determined through 10-fold cross-validation based on the minimum partial likelihood deviance, ensuring model stability and reducing overfitting.

**Validating the expression of key genes using databases**

A total of twelve genes were validated using the TCGA database and the GSE17025 dataset from the GEO database. The expression differences of these 12 genes were then analyzed in the two clusters.

**Construction of a risk model based on genes associated with TMME subtypes and validation of accuracy**

The 12 genes and their coefficients were combined to calculate a risk score for patients according to the following formula: Patients were then categorized into high- and low-risk groups based on their median risk score. Furthermore, we conducted Kaplan-Meier survival analysis (K-M survival analysis) and survival-dependent receiver operating characteristic (ROC) curve analysis for 1-, 3-, and 5-year prognostic values in the TCGA training set, and these analyses were robustly validated in the PKUPH cohort.To further prevent overfitting, the prognostic model was trained in the TCGA cohort and validated using an independent external dataset (PKUPH cohort). The concordant performance across datasets demonstrated the generalizability and robustness of the TMME-based risk model.

**Correlation of risk models with clinical characteristics**

The clinical characteristics of patients in the TCGA database and the PKUPH cohort were combined with risk stratification. The relationship between the risk score model and clinical characteristics was analyzed using the R package "pheatmap."

**Collection of somatic mutation data**

The corresponding mutation data for patients in the TCGA-UCEC cohort were obtained from the TCGA data portal (https://www.cancer.gov/tcga/). The mutational load of UCEC was determined by counting the total number of non-synonymous mutations in UCEC. The identification of UCEC driver genes was facilitated by the utilization of the "maftool" R package.

**Gene Set Enrichment Analysis of Risk Score Models and Survival Analysis of Patients in Combination with Two Subgroups**

GSEA software, provided by MsigDB, was used to determine the statistical significance of the molecular pathways as well as the concordant heterogeneity between the high- and low-risk groups. The GSEA software was provided by a Java program and downloaded from the official website (https://www.broadinstitute.org/gsea/). Statistically significant pathways were defined as those with FDR q < 0.25 and a p < 0.05. To perform a K-M survival analysis and compare survival rates, combined TMME subtyping and risk stratification were performed.

**Construction and validation of prognostic models**

The survival R software package and ggplot2 were utilized to ascertain the prognostic value of risk scores through univariate and multivariate Cox analysis. Subsequently, we employed the rms R package (v 6.7-0) and the survival R package to generate nomograms and their calibration curves. To validate the prognostic value of the risk score, we plotted Kaplan-Meier curves for the high- and low-risk groups in the TCGA-UCEC and PKUPH datasets using the survminer R software package. To assess the predictive performance of the prognostic model at 1, 3, and 5 years, we performed a ROC analysis using the pROC R software package (v 1.17.0.1) and obtained area under the curve (AUC) values and their confidence intervals for each year.

**In vivo study**

BALB/c nude mice, 5-week of age, were purchased from the animal laboratory of the People's Hospital of Peking University (Beijing, China) and raised under SPF conditions. The animal experiment was approved by the ethics committee of Peking University People's hospital, and the ethical requirements for experimental animals and the animal welfare law were strictly observed during the experimental operation. The BALB/c nude mice were divided into two groups, including the glucose metabolism disorder group (GMD group) and the non-glucose metabolism disorder group (NC group). Initially, the GMD group was administered intraperitoneal injections of streptozotocin (1% w/V solution in fresh cold sodium citrate buffer, pH 4.5), followed by a high-fat diet. In contrast, the mice in the control group were administered an equal volume of citric acid buffer and normal feed. The blood glucose levels of the test subjects were then detected by blood sugar device. The modeling was deemed successful when any random blood glucose concentration exceeded 11.1 mmol/L. Ishikawa cells (100 μL, containing approximately 3 × 106 cells) were inoculated subcutaneously in nude mice to construct two distinct groups: Ishikawa cell-loaded dysglycemic BALB/c mice and loaded non-dysglycemic BALB/c mice. Tumor size was measured at three-day intervals, and tumor volume was calculated using the formula v = a × b^2^ × 0.5, where a represents the long diameter of the tumor and b represents the short diameter of the tumor. At the conclusion of the experiment, the nude mice were euthanized, and the subcutaneous tumors and nude mouse uteri were surgically removed, photographed, and documented. In addition, the tumor tissue samples were preserved using various methods, including freezing and fixation, for subsequent studies.

**HE staining of mouse uterus**

Paraffin sections intended for staining were prepared in advance. Two deparaffinization treatments were performed using xylene. Subsequently, the samples were rehydrated through a series of ethanol washes, beginning with 100%, 95%, 85%, and 75% ethanol for five minutes each. This was followed by two washes in distilled water. The nuclei were then stained with hematoxylin for 3 minutes, after which the excess color was removed by washing with distilled water. The cytoplasm was differentiated with hydrochloric acid in ethanol solution for 30 seconds, after which it was washed twice with tap water. The cytoplasm was then stained with eosin for two minutes, after which the floating color was washed with distilled water. Following this, the slices were dehydrated with 75% ethanol, 85% ethanol, 95% ethanol, and 100% ethanol for one minute each, and then passed through xylene twice. Finally, the slices were sealed, observed, and photographed under the microscope.

**Immunohistochemical staining**

Immunohistochemical staining of tumor tissues from mice was performed. Paraffin sections were deparaffinized. Antigen repair was then performed using the autoclave repair method. The antigen repair solution (pH 6.0 citric acid) was introduced into a pressure cooker and subjected to high-temperature boiling. Sections were then immersed in 3% H2O2 for 30 minutes to deactivate endogenous peroxidase, followed by thorough washes in PBS for five minutes on three separate occasions. The next step involved the addition of rabbit anti-COL I antibody (1:300), rabbit anti-COL III, rabbit anti-HAS, rabbit anti-LAMB1, and rabbit anti-Vimentin (1:400) to the different sectioned sections. These were then placed in a wet box at 4°C overnight. The following day, after rewarming for 30 minutes, the sections were rinsed again with PBS for 5 minutes, and this step was repeated three times. HRP-labeled goat anti-rabbit IgG was then added to the sections, and the samples were incubated at room temperature for 30 minutes. They were subsequently washed three times with PBS for 5 minutes each. Subsequently, DAB solution was added to the tissues, and color development was observed under the microscope. To prevent further staining, the slides were immersed in water. Sections were then restained with hematoxylin and differentiated with a solution of ethanol hydrochloride. The slides were then dehydrated with 100% ethanol, sealed, observed under the microscope, scanned, and photographed.

**Western Blotting**

The total proteins in Ishikawa cells treated with different concentrations of glucose were extracted with RIPA buffer containing 1% PMSF (Beyotime) and quantified by BCA method (Beyotime). Western blotting was performed by taking 20 μg of protein against the main components of the cell matrix (α-SMA, COL I, COL III, HAS3, LAMB1, Vimentin).

**Cell Culture and Specialty Treatments**

The Ishikawa cell line was maintained in the Department of Obstetrics and Gynecology at the People's Hospital of Peking University (Beijing, China). The cells were cultivated in a DMEM/F12 medium containing 10% FBS at 37°C within a 5% CO_2_ incubator. Upon achieving 80-90% cell fusion, the cells were digested with 0.25% trypsin and subsequently passaged for further animal experimentation. The human endometrial stromal cell line, HESC, was cultivated in DMEM/F12 medium containing 10% CCS, 1% ITS, and 1% double antibody at 37°C in a 5% CO_2_ incubator. When the cell fusion reached 60-70%, the HESC cells were treated with different concentrations of glucose solution for 48 hours. Thereafter, the HESC cells were incubated in a 5% CO_2_ incubator at 37°C.

**Statistical analysis**

The data are expressed as the mean ± standard deviation (SD). The analysis was conducted using Graph Pad Prism 8. The Student's t-test, one-way ANOVA, Kaplan-Meier method, and chi-square test were utilized in the analysis. Bioinformatics analysis was performed using R software. Statistically significant differences were considered as those with p < 0.05 and p < 0.01.
