# Supplementary figures and images for: Integrated analysis of tumor mechanical microenvironment-based signature reveals prognostic risk and immune landscape in endometrial carcinoma
Source: Genes Dis. 2026 Jan 13;13(6):102039. doi: 10.1016/j.gendis.2026.102039 (PMC13380096; doi:10.1016/j.gendis.2026.102039)

A

Cluster Dendrogram

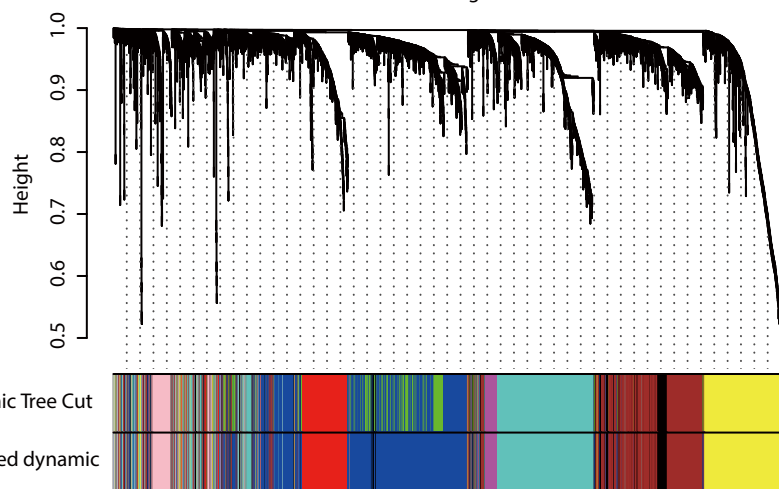

B

Module-trait relationships

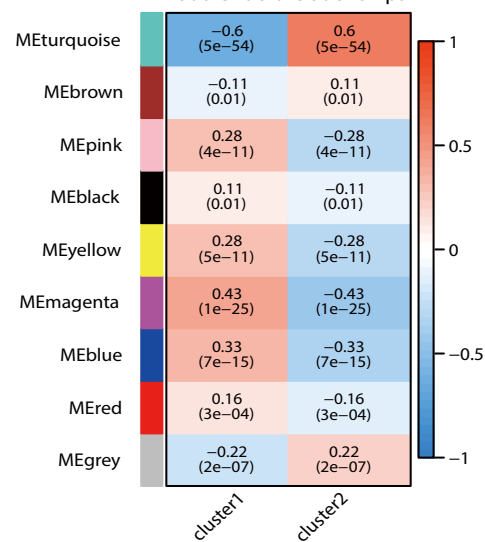

C

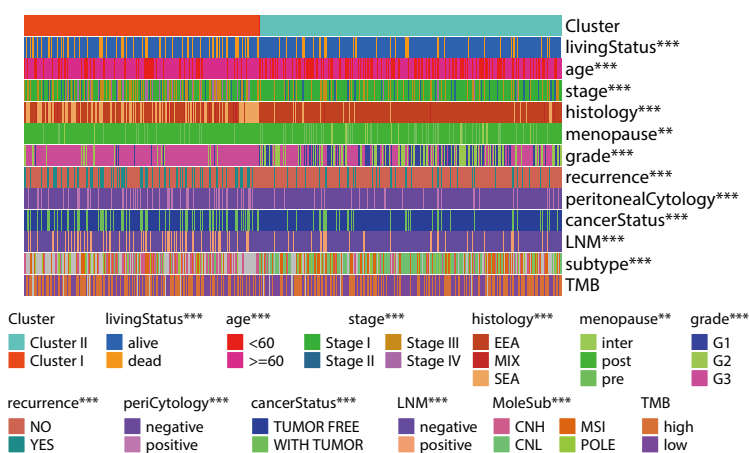

D

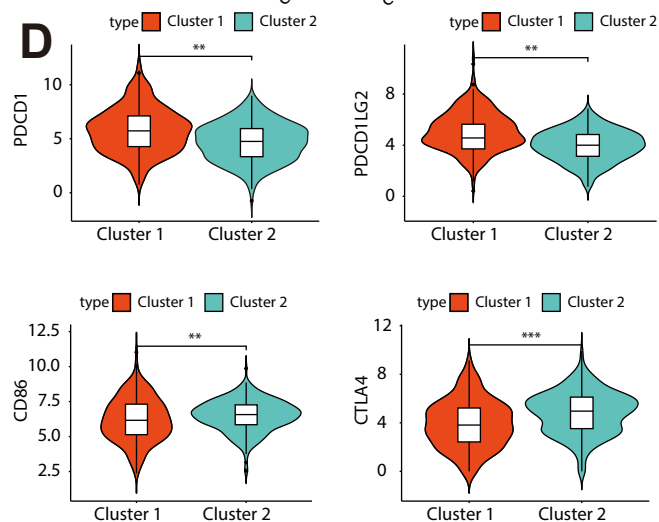

Supplement: Multimedia component 3 [file mmc3.pdf]

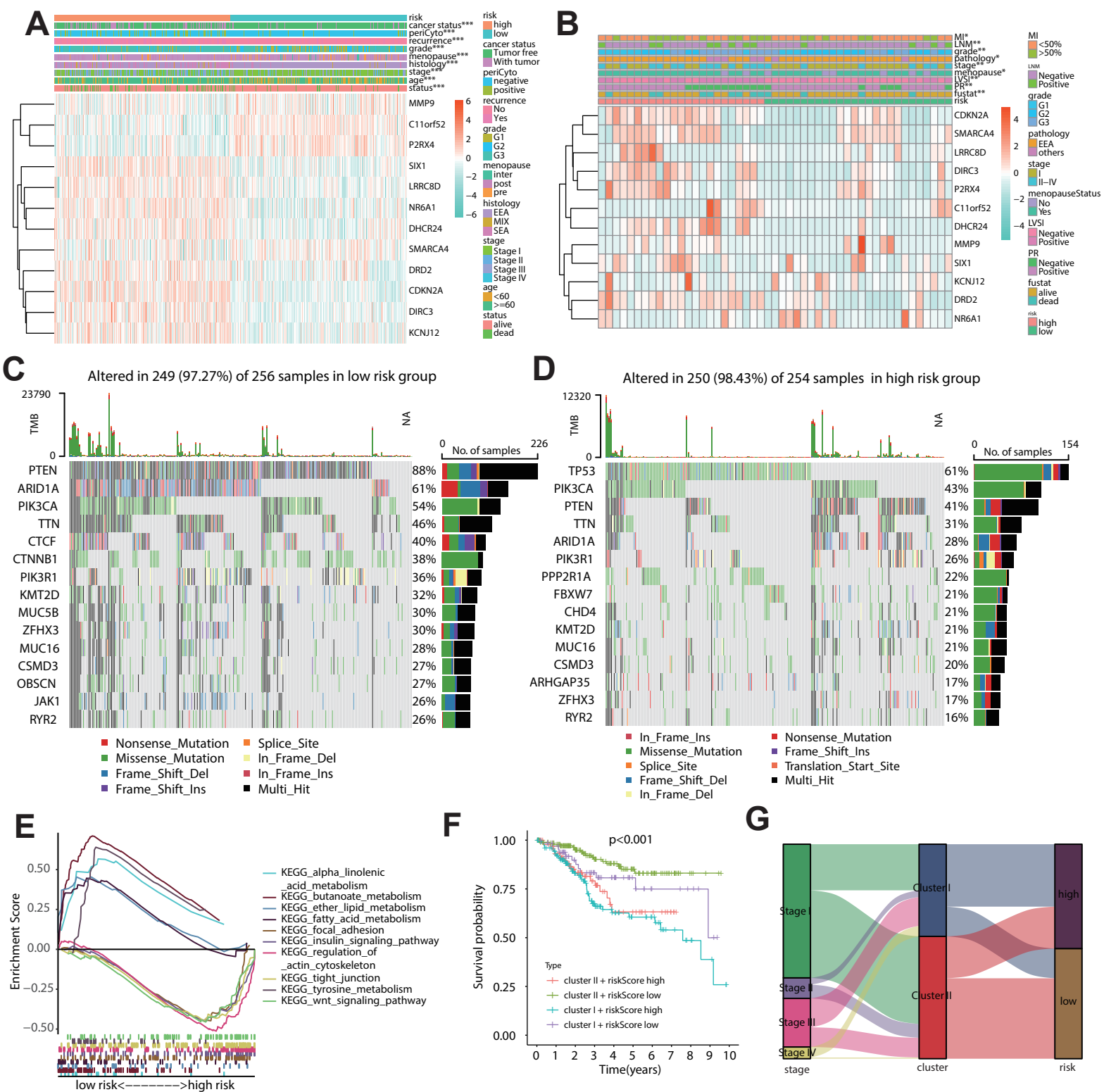

Supplement: Multimedia component 4 [file mmc4.pdf]

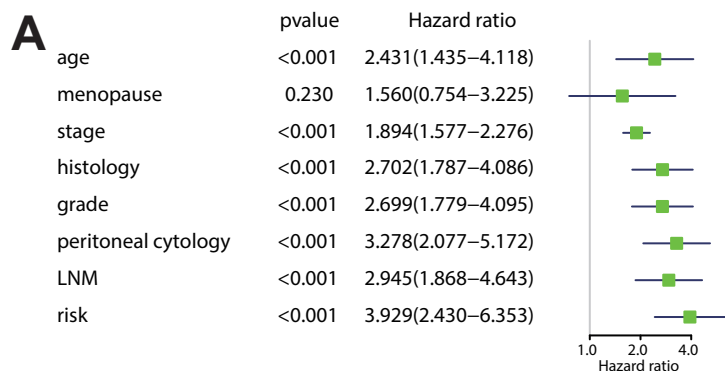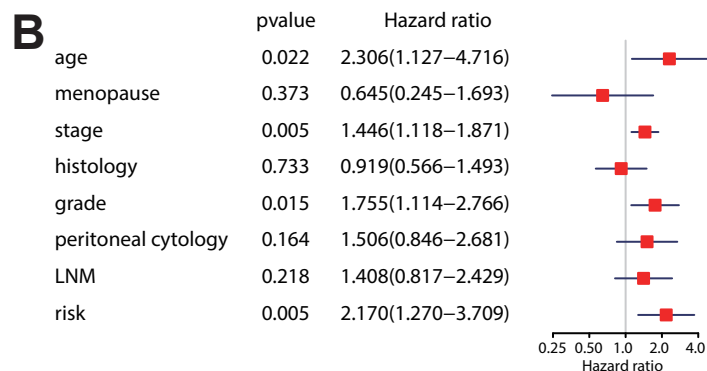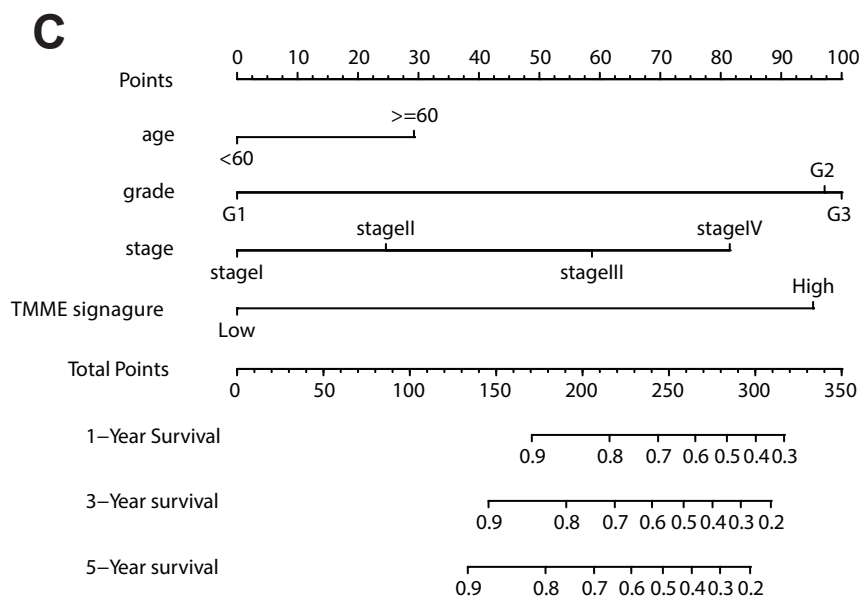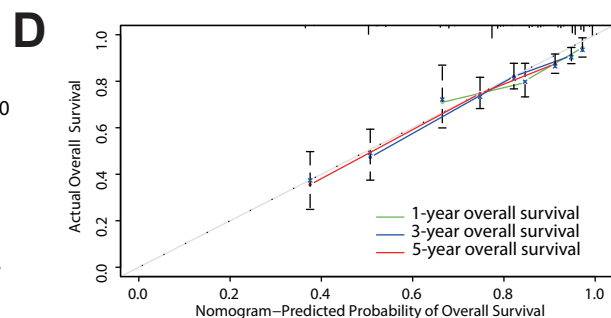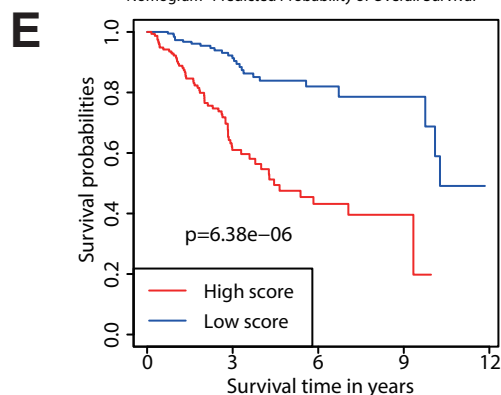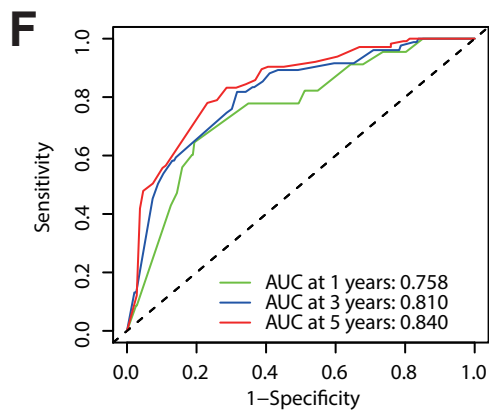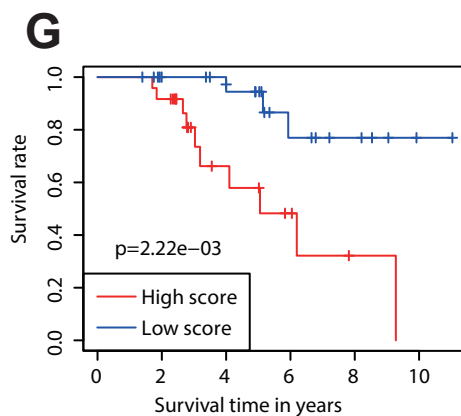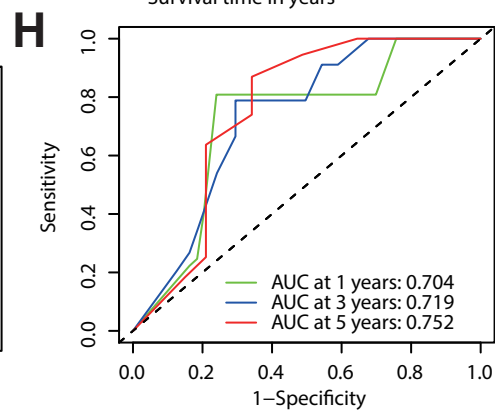

Supplement: Multimedia component 5 [file mmc5.pdf]

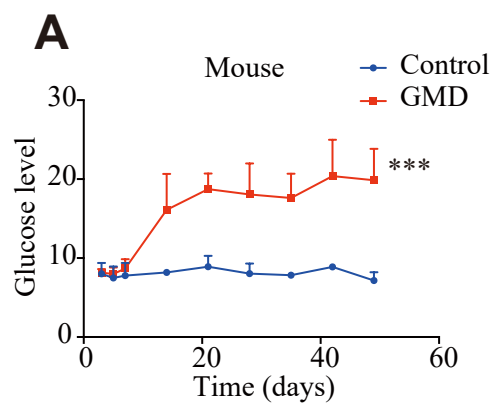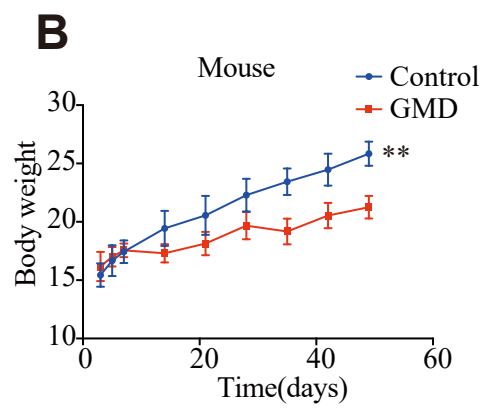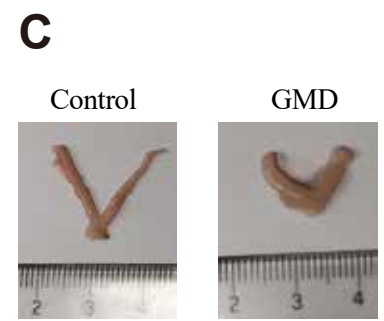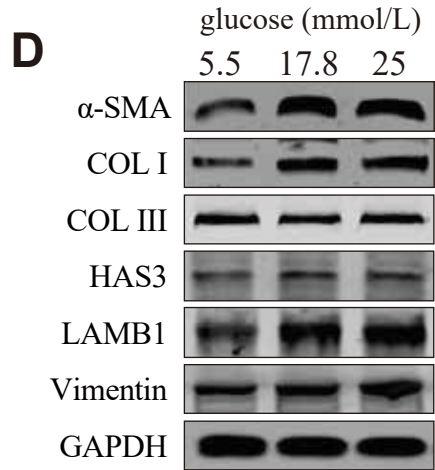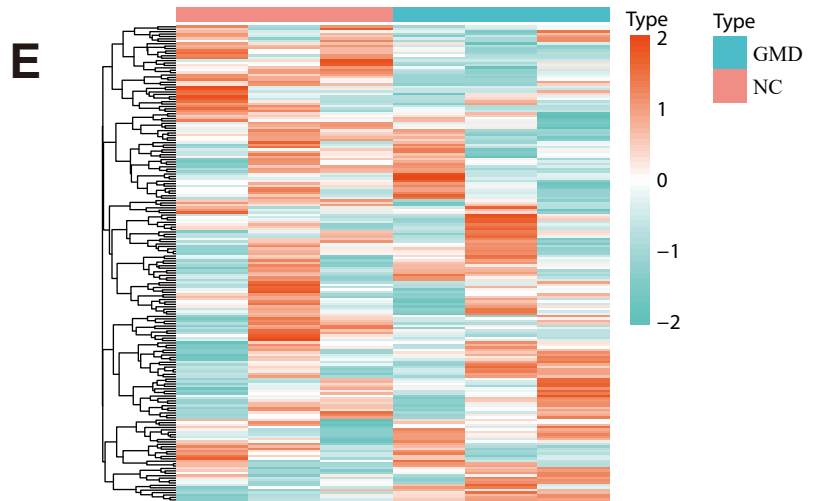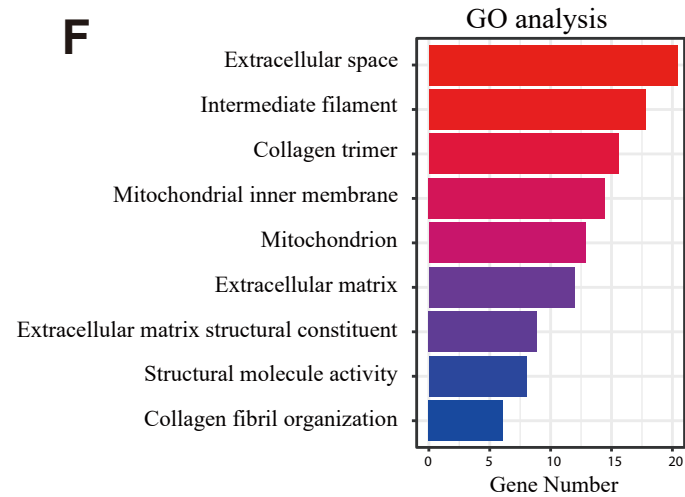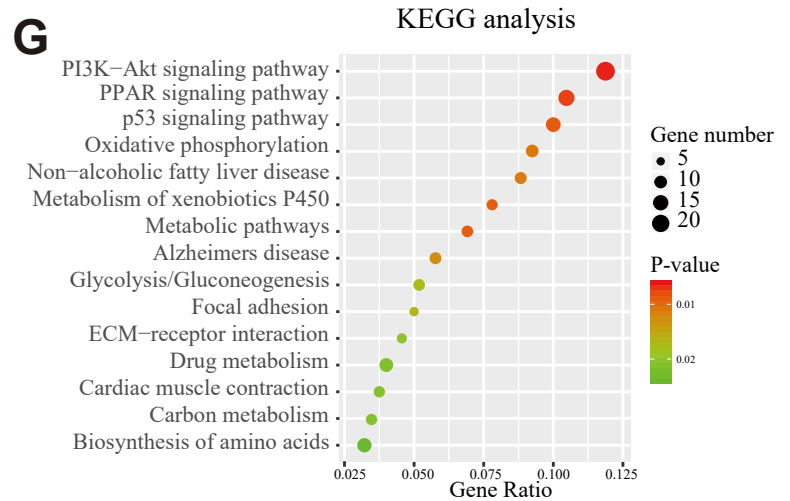

Supplement: Multimedia component 6 [file mmc6.pdf]
